# Meningoencephalitis of unknown origin in dogs under veterinary referral care in England (2017–2021): a multicenter case control study

**DOI:** 10.3389/fvets.2025.1710593

**Published:** 2025-11-26

**Authors:** Yeonsoo Choi, Dan G. O’Neill, Edward Ives, Thomas Harcourth Brown, Bruno A. Lopes, Lisa Cardoso Alves, Thomas Cardy, Marco Ruggeri, Anna Tauro, Giunio Bruto Cherubini, Mark Lowrie, Louisa Saunders, Nicolas Granger, Maria Stefaniuk, Rodolfo Cappello, Raquel Trevail, Sophie Adamantos, Rita Gonçalves, Steven De Decker, Angela Fadda

**Affiliations:** 1 Langford Vets - Bristol Veterinary School, University of Bristol, Bristol, United Kingdom; 2 Pathobiology and Population Sciences, The Royal Veterinary College, Hatfield, United Kingdom; 3 Anderson Moores Veterinary Specialists, Winchester, United Kingdom; 4 Southfields Veterinary Specialists, Basildon, United Kingdom; 5 Department of Veterinary Medicine, University of Cambridge, Cambridge, United Kingdom; 6 Cave Veterinary Specialists, Wellington, United Kingdom; 7 ChesterGates Veterinary Specialists, Chester, United Kingdom; 8 Access Specialty Animal Hospital, Pasadena, CA, United States; 9 Dick White Referrals, Cambridge, United Kingdom; 10 Department of Veterinary Sciences, University of Pisa, Pisa, Italy; 11 Movement Referrals: Independent Veterinary Specialists, Uttoxeter, Staffordshire, United Kingdom; 12 Highcroft Veterinary Referrals, Bristol, United Kingdom; 13 North Downs Specialist Referrals, Bletchingley, United Kingdom; 14 Southern Counties Veterinary Specialists, Ringwood, United Kingdom; 15 Paragon Veterinary Referrals, West Yorkshire, United Kingdom; 16 School of Veterinary Science, University of Liverpool, Liverpool, United Kingdom; 17 Royal Veterinary College, London, United Kingdom

**Keywords:** canine, encephalitis, demographic risk, case control, meningoencephalitis

## Abstract

**Background:**

A considerable body of published research on meningoencephalitis of unknown origin (MUO) exists; however, certain fundamental aspects relating to the epidemiology remain poorly characterized. These include the frequency of MUO diagnosis at referral level, reliable demographic risk factors, and the extent to which proposed diagnostic criteria are applied in referral clinical settings.

**Methods:**

Based on 1,121 MUO cases (from 01 January 2017 to 31 December 2021) treated at 13 referral centers in England and 750,000 control dogs from the VetCompass Programme, this study investigated a range of demographic risk factors using multivariable logistic regression analysis. Additionally, we report on clinical methods used for diagnosis.

**Results:**

Meningoencephalitis of unknown origin represented 2.21% (95% CI: 2.08–2.34) of new neurological referrals (1,121/50721). Clinical diagnosis included both magnetic resonance imaging (MRI) and cerebrospinal fluid (CSF) analysis in 1026 (91.5%) cases. Of these, 961 (89.3%) showed results indicative of MUO in both MRI and CSF. Abnormal MRI but normal CSF were found in 55 cases (5.4%), while normal MRI and abnormal CSF were found in 36 cases (3.5%). Both normal MRI and normal CSF were reported in 19 cases (1.5%). Screening for infectious disease was carried out in 1037 (92.5%) of cases. The diagnosis of MUO peaked at 4 years (median 4.33, IQR 2.50–6.92, range 0.30–15.00) and declined after age 10. Welsh Springer Spaniel (OR 23.76, 95% CI 10.37–54.43), Maltese (OR 20.53, 95% CI 14.53–29.01), Papillon (OR 17.48, 95% CI 7.66–39.91), Boston Terrier (OR 17.31, 95% CI 11.17–26.82), and French Bulldog (OR 9.14, 95% CI 7.14–11.71) had the highest MUO odds compared to crossbreed dogs. Brachycephalic breeds had 2.56 times higher odds (95% CI 2.23–2.95) than mesocephalic breeds. Dogs ≥15 kg had lower odds than those <10 kg.

**Conclusion:**

This study provides the largest referral-based analysis of MUO cases to date, offering updated insights into breed predispositions and clinical diagnosis. This more precise characterization of the demographic factors adds valuable context for future research design, particularly in breed-focused investigations and risk stratification. By documenting current diagnostic practices used by referral specialists, this work lays the foundation for greater consistency in case recognition and offers practical guidance for structuring future MUO clinical trials.

## 1 Introduction

Meningoencephalitis of unknown origin (MUO) is an umbrella term to indicate a presumed autoimmune inflammatory disease of the central nervous system (CNS) in dogs (1, 2). Despite decades of research (1–39) many aspects of MUO remain poorly understood, resulting in persistent knowledge gaps that continue to hinder scientific and welfare progress.

First, an accurate estimation of MUO prevalence across the general canine population is currently unattainable. This is primarily due to the diagnostic complexity involved, requiring advanced investigations, including neuroimaging and cerebrospinal fluid (CSF) analysis, which are not routinely accessible in primary care settings. As a result, more definitive diagnoses are largely limited to referral-level institutions, introducing an inherent selection bias when reporting using only referral data. Reported frequencies of MUO at referral level vary across the literature, likely due to differences in case definition, diagnostic criteria, and population demographics among studies. An incidence between 5 and 25% of all CNS canine disorders was reported for the granulomatous meningoencephalitis (GME), subtype of MUO, in early studies (35–37), while in others, GME accounted for more than 55% of all encephalitis diagnosed in European and American veterinary teaching hospitals (23, 40). More recently, an estimated 47.5% of dogs presenting to two large referral institutions in the UK with signs of CNS inflammation were diagnosed with MUO (41). This heterogeneity limits comparability and hinders consistent disease mapping across studies, thereby complicating the development of effective research strategies, particularly the design of clinical trials, where adequate sample sizes and homogeneous inclusion criteria are required (42).

Second, diagnosis of MUO remains presumptive in most cases and relies on a combination of inference from signalment, clinical, imaging findings and CSF analysis. However, since no internationally recognized standard for antemortem diagnosis of MUO exists, classification may differ among institutions and specialists. The importance of conducting robust clinical trials for MUO treatments has been emphasized (13, 43), with proposed eligibility criteria for MUO therapeutic trials previously published (13), although their application in clinical practice has not been systematically evaluated. The potential of low adherence to these criteria could indicate that further refinement is needed to ensure broader clinical relevance and improve the generalizability of study findings.

Third, the aetio-pathogenesis of MUO remains elusive. Although genetic predisposition and immunological triggers are suspected (2, 22), their relative contribution remains unclear. Previous studies have examined predispositions for two MUO subtypes -granulomatous meningoencephalitis (GME) and necrotizing meningoencephalitis (NME) - across breeds and age groups (5, 8, 20, 21, 23, 24, 34, 44). It has been historically reported that NME primarily affects young dogs under 4 years, with breed-specific trends observed particularly in toy and small breeds such as Pugs, Yorkshire Terriers, Maltese, Chihuahuas, Pekingese, Papillons, and various other pure and mixed breeds (1, 13, 32). In contrast, GME tends to present in middle-aged dogs, most notably among toy and terrier breeds, including Maltese, West highland White Terrier, Miniature poodles, aged between 4 and 8 years (1, 13, 21). Regarding sex, while early literature suggested a female predominance in GME (8, 9, 45), more recent studies have not supported any significant sex effect across MUO cases. Importantly, although demographic patterns are reported, MUO is not exclusive to certain age groups or breeds; dogs of any age, size, or breed, including large breed dogs over 15 kg (3, 28) can develop the condition. It is unclear, however, whether these reported patterns are truly representative of the wider canine population, as existing data are largely derived from small-scale studies within secondary care settings, raising concerns about selection bias (13, 21). Gaining a more precise understanding of demographic influences, such as age, breed, and sex, could aid in unravelling disease mechanisms and optimizing future research — for instance, by identifying breed-associated genetic variants or highlighting environmental risk factors in need of further investigation.

In order to fill some of these critical information gaps, the current study aimed to estimate the frequency of MUO diagnosis at referral level in UK, to provide an overview of diagnostic approaches currently used by veterinary specialists, and to investigate demographic characteristics and risk factors using a large population of dogs attending primary veterinary care in the UK as a comparator baseline. Breed effects were a factor of primary interest for the risk factor analysis.

## 2 Materials and methods

### 2.1 Study populations and data source

Medical records from the Neurology and Neurosurgery services of 13 of referral centers across England were reviewed for the period spanning 1 January 2017 to 31 December 2021. The review aimed to identify all dogs with a diagnosis of MUO (case population), as well as the total number of dogs referred to these services during the same timeframe (neurology referral case load population).

Cases with a diagnosis of MUO (case population) were either retrieved by a board-certified neurologist or a supervised resident/intern of participating practices and provided as summary data, or by direct review of their clinical records by one of the authors (YC). Cases were included if they had been diagnosed with MUO by a neurology specialist and met one or more of the following criteria: (1) Neurological examination localizing to the forebrain, brainstem, cerebellum or multifocal with a history compatible with inflammatory brain disease (acute or subacute onset, multifocal neurological signs, and progressive course), (2) MRI findings compatible with inflammatory brain disease, or a normal brain MRI in combination with an abnormal CSF (consistent with mononuclear CSF pleocytosis), (3) Abnormal CSF or normal CSF in combination with an abnormal MRI compatible with inflammatory brain disease, or (4) Normal MRI and CSF findings with a compatible presentation and progression of signs. Cases were excluded when an active CNS infection by *Neospora caninum* and *Toxoplasma gondii* was confirmed. For each MUO case the demographic data (breed, sex, neuter status, date of presentation, and age at presentation and weight, when available) and diagnostic data (investigations performed, MRI findings, CSF findings, infectious disease screening results) were recorded. If any diagnostic tests had not been performed, reasons stated for not performing the test were also recorded when available.

A separate control group (control population) consisting of 750,000 dogs under primary veterinary care at practices participating in the VetCompass Programme in 2019 (46) was used for demographic risk factor analysis. The year 2019 was selected to offer a temporally comparative primary care dog population approximately midway across the date range for the referral MUO cases. Ethical approval for the use of the referral data was obtained from the Langford Veterinary services – University of Bristol ethical approval committee of (VIN/22/004) and for the use of the VetCompass data from the Social Science Research Ethical Review Board at the Royal Veterinary College (URN SR2024-01632811).

Demographic and clinical data on the cases and controls were compiled into a single commercial computer database (Microsoft Excel).

### 2.2 MUO incidence risk

The incidence risk of newly diagnosed MUO cases over the five-year observation period was calculated from the total number of MUO cases as a proportion of the total number of cases admitted to all 13 neurology referral services. Yearly incidence risks were calculated by dividing the annual number of newly diagnosed MUO cases from all 13 referral centers by the annual number of referred canine neurological cases in each respective year (Table 1). It should be noted, however, that three of the 13 referral centers commenced operations after 2019 and therefore did not contribute data to the 2017–2019 totals. Despite this, all yearly incidence risks were calculated using the combined data available from all 13 centers over the entire study period. To enable center-specific assessment, yearly incidence risks were also calculated separately for each referral center (Table 2). Ninety-five percent confidence intervals (CIs) for incidence estimates were calculated according to previously published methods (47).

**Table 1:** Yearly incidence of MUO among neurological cases from 2017 to 2021, presented as absolute numbers and percentages.

Year	Muo cases: No. (%)	MUo non cases: No. (%)	Total neuro cases
2017	215 (2.56)	8,196 (97.44)	8,411
2018	189 (2.13)	8,695 (97.87)	8,884
2019	224 (2.26)	9,699(97.74)	9,923
2020	242 (2.08)	11,411(97.92)	11,653
2021	251(2.12)	11,599 (97.88)	11,850

**Table 2:** Yearly incidence risk (%) of MUO in dogs, with corresponding 95% confidence intervals, calculated for each of the 13 referral centers in England over the period 2017–2021.

	Yearly incidence risk of MUO (2017–2021) (%)					95% Confidence interval (CI)				
Referal centeres	2017	2018	2019	2020	2021	2017	2018	2019	2020	2021
A	5.17	3.55	2.31	3.07	2.72	3.60–7.37	2.36–5.32	1.41–3.78	1.99–4.69	1.77–4.17
B	4.76	2.87	3.14	4.18	3.02	2.53–8.80	1.32–6.12	1.53–6.34	2.29–7.53	1.60–5.64
C*	–	–	–	1.27	2.07	–	–	–	0.73–2.20	1.36–3.15
D	1.14	1.64	5.09	1.21	2.44	0.31–4.05	0.29–8.72	2.87–8.89	0.56–2.61	1.33–4.44
E	1.43	0.94	0.41	0.65	0.48	0.98–2.08	0.60–1.46	0.21–0.77	0.40–1.05	0.27–0.87
F	3.77	4.32	5.06	4.19	4.72	2.37–5.95	2.84–6.52	3.63–7.02	2.85–6.11	3.24–6.82
G**	–	1.13	1.28	1.05	0.88	–	0.31–4.03	0.95–2.77	0.45–2.45	0.40–1.90
H	1.34	1.81	2.63	1.84	1.56	0.68–2.62	1.10–2.97	1.76–3.92	1.13–2.96	0.93–2.59
I**	–	5.56	5.20	3.99	3.88	–	2.57–11.59	3.06–8.69	2.47–6.38	2.58–5.80
J	2.65	1.93	2.55	2.15	2.02	1.95–3.59	1.36–2.74	1.87–3.47	1.54–3.01	1.41–2.89
K	1.30	1.52	2.44	2.32	1.48	0.75–2.26	0.87–2.64	1.58–3.74	1.52–3.53	0.85–2.57
L	3.65	2.80	2.15	2.53	1.63	2.52–5.26	1.82–4.28	1.28–3.57	1.61–3.96	0.95–2.76
M	3.14	2.67	2.45	3.24	4.21	2.36–4.17	1.92–3.68	1.76–3.41	2.42–4.32	3.27–5.40
Total	2.56	2.13	2.26	2.08	2.12	2.24–2.92	1.85–2.45	1.98–2.57	1.83–2.35	1.87–2.39

* Referral center C started neurology service since 2020. ** Referral centre G and I started neurology service since 2018.

To determine whether the yearly incidence risk of MUO changed significantly from 2017 to 2021, a chi-square test for homogeneity was performed using GraphPad Prism (version 2025). Frequencies of MUO cases across the 5 years were compared, and statistical significance was set at *p* < 0.05.

To assess whether the seasonal distribution of MUO cases differed significantly, a chi-square goodness-of-fit test was performed comparing observed frequencies across four seasons (spring, summer, autumn, winter) with expected frequencies under the assumption of a uniform distribution. A *p*-value < 0.05 was considered statistically significant.

### 2.3 Evaluation of MUO diagnostic criteria

**Figure 1:**
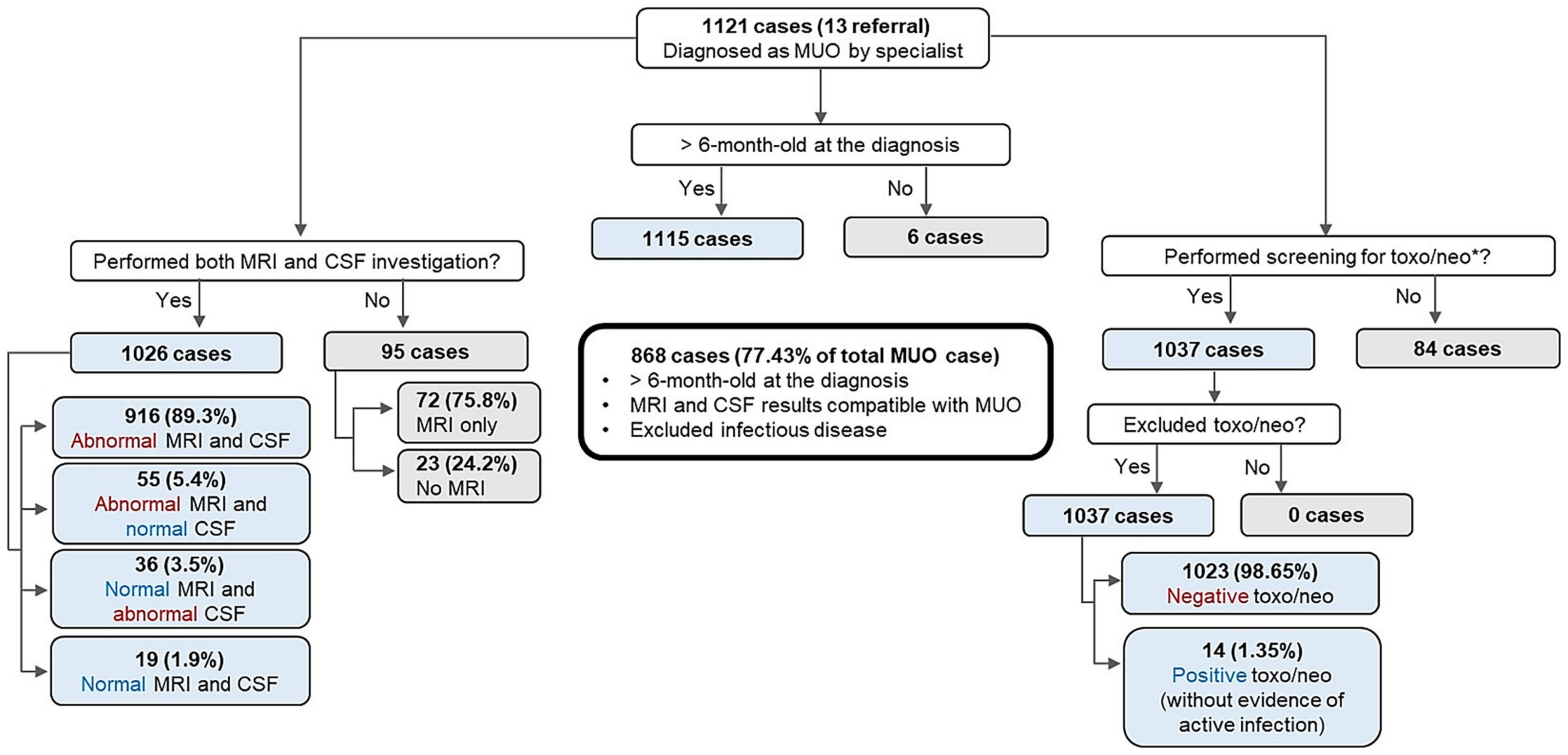
Classification of MUO cases according to investigation methods and outcomes. MRI (magnetic resonance imaging), CSF (cerebrospinal fluid), *toxo/neo (*Toxoplasma gondii* and *Neospora caninum* screening). The central box with thicker borders highlights the subset of dogs potentially eligible for clinical trials, according to Granger et al.

Eligibility criteria for recruiting cases of MUO into therapeutic clinical trials, where histopathological confirmation is unavailable, were proposed by Granger et al. (13) based on a systematic review of 457 published cases. These criteria include (1) dogs to be older than 6 months of age at the time of diagnosis; (2) showing multiple, single or diffuse intra-axial hyperintensities on T2-weighted (T2W) MR images; (3) monocytic pleocytosis on CSF analysis (with >50% of monocytes/ lymphocytes;) and (4) exclusion of active infections commonly occurring in the specific geographic area. To evaluate how frequently these criteria were met when making a diagnosis of MUO for the current study dogs, and thereby estimate how many of the currently collected cases would have been potentially eligible for inclusion in a clinical trial using these criteria, the current MUO cases were categorized as follows: MRI performed (yes/no); MRI changes consistent with the above criteria (MRI abnormal) or not (MRI normal); CSF analysis performed (yes/no); CSF changes consistent with the above criteria (CSF abnormal) or not (CSF normal); infectious disease testing performed (yes/no), and the infectious agents tested. Reasons for test omission were recorded and grouped into categories generated after review. Descriptive statistics were used to present case proportions, determine how frequently the diagnostic criteria were applied, which tests were most and least often used, and the rationale behind their omission (Figure 1).

### 2.4 Risk factor analysis

Risk factor analysis used a case–control study design to explore associations for a range of demographic risk factors for an outcome of MUO. The cases included the 1,121 referral dogs as MUO case and a random sample of 750,000 dogs under primary veterinary care at clinics participating in the VetCompass Programme during 2019 with no evidence of MUO in their clinical records as controls, in line with methods previously reported (48). VetCompass dogs under veterinary care were defined as dogs with ≥1 electronic health record (EHR) (free-text clinical note, treatment or bodyweight) recorded during 2019. VetCompass collates de-identified EHR data from primary-care veterinary practices in the UK for epidemiological research (VetCompass 2024). Data fields for each animal include fixed values for species, breed, date of birth, sex and neuter status along with date-specific information on clinical notes, bodyweight and treatment. The overall list of 2,250,417 VetCompass dogs under veterinary care in 2019 was searched across all dogs for the terms mening* or MUO in all available clinical notes at any date up to 31 December 2023. After removing the 11,607 dogs identified with at least one of these terms, a random sample of 750,000 VetCompass dogs was selected as controls for the risk factor analysis from the remaining pool of 2,238,810 dogs.

Breed descriptive information entered by the participating practices for both the cases and controls was cleaned and mapped to a VetCompass breed list derived and extended from the VeNom Coding breed list that included both recognized purebred breeds and designer crossbreed breed terms (The VeNom Coding Group 2024). A breed purity variable categorized all dogs of recognizable breeds as ‘purebred’, dogs with contrived names generated from two or more purebred breed terms as ‘designer crossbreed’ crossbreds (purposely bred crossbreeds) and dogs recorded as mixes of breeds but without a contrived name as ‘general crossbred’ (The Kennel Club 2024). A breed variable was developed that included individual pure breeds and designer hybrids represented by ≥5 MUO cases or ≥4,000 dogs in the overall risk factor analysis dataset of 751,121 dogs, along with a single grouping of all remaining dogs with a recognizable breed and also a single grouping of all general crossbred dogs. This approach was taken to facilitate statistical power for the individual breed analyses (49).

All breeds were further characterized to develop additional derived variable on skull shape (dolichocephalic, mesocephalic, brachycephalic, uncategorized), and brachycephalic severity (mild, moderate and severe) (50). A ‘Kennel Club recognized breed’ variable categorized breeds as recognized by the Kennel Club or not. A ‘Kennel Club breed group’ variable classified breeds recognized by the UK Kennel Club into their relevant breed groups (Gundog, Hound, Pastoral, Terrier, Toy, Utility and Working) and all remaining types were classified as non-Kennel Club recognized (The Kennel Club 2024).

A sex-neuter variable (female entire, female neutered, male entire, male neutered, unrecorded) described the recorded status at the date of MUO diagnosis for the cases and at the final available EHR value for the VetCompass controls.

Adult bodyweight was calculated for all 2,250,417 VetCompass dogs with relevant data as the median of all bodyweights (kg) values recorded for each dog after reaching 18 months old. Bodyweight data were not available for all the MUO cases, so adult bodyweight was imputed for each MUO case based on the relevant VetCompass value for that breed and sex. Adult bodyweight (kg) was categorized as: <10.0, 10.0 to <15.0, 15.0 to < 20.0, 20.0 to < 25.0, 25.0 to < 30.0, 30.0 to < 40.0, and ≥ 40.0. Age (years) was defined at the date of MUO diagnosis for the case populations, and at December 31, 2019 for the VetCompass controls. Age was categorized in one-year bands to 14 years.

The referral centers sharing data on the MUO cases and the veterinary groups sharing data on the VetCompass controls were anonymized and included in the analysis as a fixed effect to account for confounding.

Following internal validity checking and data cleaning, analyses were conducted using Stata Version 16 (Stata Corporation). Results for clinical management were reported descriptively. Normality of distributions were evaluated by assessing a histogram. For non-normal distributions, descriptive results reported the median, interquartile range (IQR) and range, and univariable binary comparison used the Mann–Whitney test.

Breed-specific age distributions were summarized separately for MUO cases and the VetCompass control population. For both MUO cases and VetCompass controls, descriptive statistics (mean, median, IQR and range) were calculated per breed and used to evaluate whether MUO diagnosis occurred at comparable ages to the general breed population or deviated from demographic expectations (Supplementary File S1).

Univariable analysis of categorical variables used the chi-square test (47). Risk factor analysis used binary logistic regression modelling to evaluate univariable associations between risk factors (breed, breed purity, Kennel Club recognized breed, Kennel Club breed group, skull shape, brachycephalic severity, spaniel type, adult bodyweight, age, sex-neuter, veterinary group) and an outcome of MUO diagnosis. Because breed was a factor of primary interest for the study, variables derived from, or related to, the breed information and therefore highly correlated with breed (breed purity, Kennel Club recognized breed, Kennel Club breed group, skull shape, brachycephalic severity, spaniel type, adult bodyweight) were excluded from initial breed multivariable modeling. Instead, each of these excluded variables individually replaced the breed variable in the main breed-focused model to evaluate their effects after taking account of the other variables, as previously described (51). Risk factors with liberal associations in univariable modelling (*p* < 0.2) were taken forward for multivariable evaluation. Model development used manual backwards stepwise elimination. Pair-wise interaction effects were evaluated for the final model variables (52). The area under the Hosmer-Lemeshow test, ROC curve and McKelvey and Zavoina Pseudo-*R*^2^ were used to evaluate the quality of the model fit (52, 53) Statistical significance was set at *p* < 0.05.

## 3 Results

### 3.1 Incidence and diagnosis of MUO at referral level

During the study period (2017–2021), 23 referral centers in England were contacted. Four centers declined to participate, four did not respond, and two later withdrew, leaving clinical data submitted from 13 centers in the final analysis. Data on the numbers of MUO cases diagnosed and total number of dogs referred to each neurological service were available for all 13 centers. Data were available from 2017 for all referral centers except three centers that commenced operations in either 2018 or 2020. Six centers provided summary data (496 cases), while for seven centers data were extracted by review by one of the authors (YC) (625 cases). The study included 1,121 MUO cases diagnosed between 1 January 2017 and 31 December 2021 from a total of 50,721 dogs referred to the 13 contributing veterinary neurology specialistic services. This equated to an overall incidence risk of 22 new MUO cases from every 1,000 neurological cases referred to a veterinary specialist, or an incidence risk of 2.21% (95% CI, 2.08–2.34) of MUO cases.

The annual incidence risk of MUO from 2017 to 2021 did not show any significant changes over the observational period (chi-square test for homogeneity p-value = 0.265). In 2017, 215 MUO cases were diagnosed among 8,411 dogs referred for neurological care (2.56%; 95% CI: 2.24–2.92); in 2018, 189 cases among 8,884 referrals (2.13%; 95% CI: 1.85–2.45); in 2019, 224 cases out of 9,923 (2.26%; 95% CI: 1.98–2.57); in 2020, 242 cases among 11,653 (2.08%; 95% CI: 1.83–2.35); and in 2021, 251 cases out of 11,850 (2.12%; 95% CI: 1.87–2.39) (Table 1).

Overlap of confidence intervals for the annual incidence risk of MUO across years did not suggest substantial variation in incidence over time (Table 2).

From 996 MUO cases with an available date of diagnosis, there was no evidence of seasonal distribution: spring (*n* = 227, 23.50%), summer (*n* = 261, 27.02%), autumn (*n* = 225, 23.29%), winter (*n* = 253, 26.19%) (chi-square goodness-of-fit test *p*-value = 0.186).

**Figure 2:**
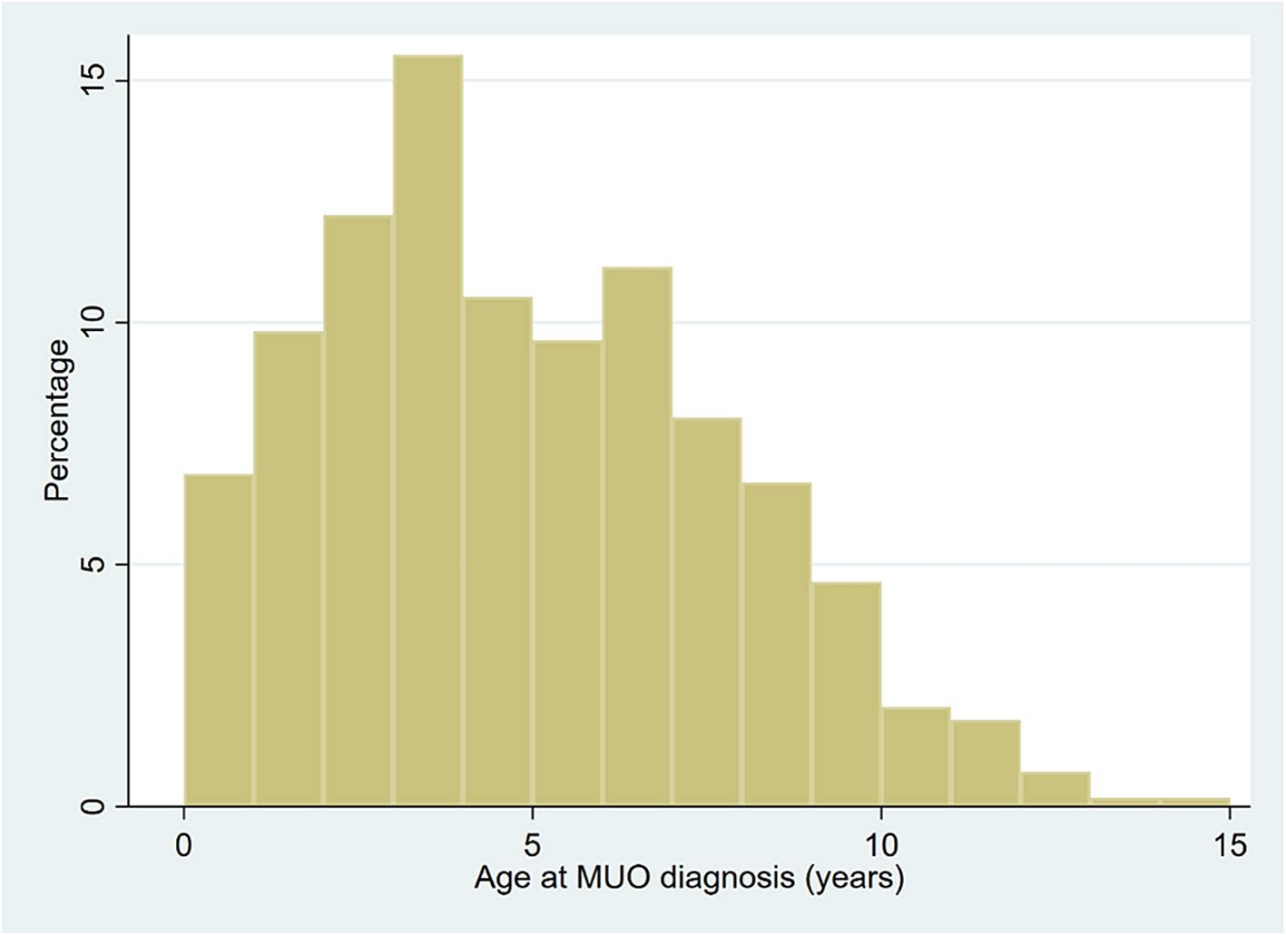
Age (years) at referral diagnosis of meningoencephalitis of unknown origin (MUO) in dogs in the UK. *N* = 1,121.

Most of the MUO cases (1,115, 99.46%), were older than 6 months at diagnosis. Median age of diagnosis for MUO cases was 4.33 years (IQR 2.50–6.92, range 0.30–15.00). The peak in ages occurred between 3.0 and 4.0 years, with 174 cases (15.5%) diagnosed in that age bracket. Overall, 749 cases (67% of the total) were diagnosed within the first 6 years of life. The number of cases declined steadily in older age brackets, with 55 cases (4.9%) diagnosed after age 10 and only 12 cases (1.1%) diagnosed in dogs aged over 12 years (Figure 2).

Overall, 1,026/1121 (91.5%) MUO cases were diagnosed using both MRI and CSF analysis (core modalities). Within this group, 916 (89.3%) showed concurrent abnormalities on MRI and CSF, 55 (5.4%) had abnormal MRI with normal CSF, 36 (3.5%) showed normal MRI and inflammatory CSF, and 19 (1.9%) were diagnosed as MUO despite normal results on both tests. Diagnostic assessments not encompassing both core modalities were recorded in 95 cases (8.5%), with 72 (75.8%) diagnosed based solely on MRI findings. Most MRI scans were performed using high-field systems. Of the 13 referral centers, 11 were equipped with 1.5 T scanners, one with a low-field 0.4 T scanner, and one center initially used a low-field scanner before upgrading to a high-field system for cases from 2019 onward. The most frequently cited reason for omitting CSF sampling was perceived risk of brain herniation; other cases were excluded due to CSF contamination or inadequate sampling. In cases diagnosed without MRI, financial limitations were the main constraint, and clinicians based their diagnosis on CSF inflammatory profiles and/or findings on computed tomography (CT) instead (Figure 1). Screening for *Toxoplasma gondii* and *Neospora caninum* infection, using mainly a combination of polymerase chain reaction (PCR) and serology, or PCR or serology alone was performed in 91.3% of the 1,121 cases.

Of the 1,121 MUO cases, 868 (77.43%) fulfilled the diagnostic criteria for eligibility in clinical trials proposed by Granger et al. (13) (Figure 1).

### 3.2 Risk factors

Risk factor analysis included 1,121 referral MUO cases diagnosed from January 1st, 2017 to December 31st, 2021 at 13 UK veterinary referral centers and a random sample of 750,000 dogs as controls with no evidence of MUO in their clinical records under primary veterinary care sourced from six large veterinary groups participating in the VetCompass Programme during 2019.

Cases with MUO were statistically significantly more likely to be purebred than the VetCompass control dogs (MUO cases 82.43% purebred vs. VetCompass controls 69.32% purebred, *p* < 0.001) (Table 3). Adult bodyweight (kg) of MUO cases was statistically significantly lower (median 10.00, IQR 7.30–13.65, range 3.40–70.00) than the VetCompass control dogs (median 13.70, IQR 8.40–24.45, range 1.39–106.00) (*p* < 0.001) (Table 4). Cases with MUO were statistically significantly younger (years) at diagnosis (median 4.33, IQR 2.50–6.92, range 0.30–15.00) than the VetCompass control dogs on December 31, 2019 (median 5.25, IQR 2.25–8.97, range 0.00–24.91) (*p* < 0.001) (Figure 2).

**Table 3:** Descriptive and univariable logistic regression results for breed-derived risk factors for meningoencephalitis of unknown origin (MUO) in dogs in the UK.

Variable	Category	Case No. (%)	Control No. (%)	Odds ratio	95% CI*	Category *p*-value	Variable *p*-value
Breed purity	General crossbred	152 (13.56)	179,231 (24.04)	Base			**<0.001**
	Designer crossbreed	45 (4.01)	49,521 (6.64)	1.07	0.77–1.49	0.684	
	Purebred	924 (82.43)	516,755 (69.32)	2.11	1.78–2.50	**<0.001**	
Kennel club recognized breed	Not recognized	201 (17.93)	242,425 (32.52)	Base			**<0.001**
	Recognized	920 (82.07)	503,082 (67.48)	2.21	1.89–2.57	**<0.001**	
Kennel club breed group	Not Kennel Club recognized breed	201 (17.93)	242,425 (32.52)	1.00			**<0.001**
	Toy	345 (30.78)	92,347 (12.39)	4.51	3.79–5.36	**<0.001**	
	Utility	244 (21.77)	88,069 (11.81)	3.34	2.77–4.03	**<0.001**	
	Hound	47 (4.19)	32,181 (4.32)	1.76	1.28–2.42	**<0.001**	
	Terrier	129 (11.51)	97,775 (13.12)	1.59	1.28–1.99	**<0.001**	
	Gundog	107 (9.55)	124,183 (16.66)	1.04	0.82–1.31	0.748	
	Working	22 (1.96)	25,768 (3.46)	1.03	0.66–1.60	0.896	
	Pastoral	26 (2.32)	42,759 (5.74)	0.73	0.49–1.10	0.137	
Skull conformation	Mesocephalic	418 (45.24)	323,358 (62.57)	Base			**<0.001**
	Brachycephalic	435 (47.08)	131,357 (25.42)	2.56	2.24–2.93	**<0.001**	
	Dolichocephalic	71 (7.68)	62,040 (12.01)	0.89	0.69–1.14	0.343	
Brachycephalic severity	Mild	26 (5.98)	15,763 (12.00)	Base			**<0.001**
	Moderate	147 (33.79)	44,648 (33.99)	2.00	1.31–3.03	0.001	
	Severe	262 (60.23)	70,946 (54.01)	2.24	1.50–3.35	**<0.001**	

Case no.: 1,121 referral MUO cases. Control no.: 750,00 dogs under UK primary veterinary care in 2019 in the VetCompass Programme. Column percentages shown in brackets. *p*-values < 0.050 are bolded. *CI confidence interval.

**Table 4:** Descriptive and univariable logistic regression results for non-breed-related demographic risk factors for meningoencephalitis of unknown origin (MUO) in dogs in the UK.

Variable	Category	Case No. (%)	Non-case No. (%)	Odds ratio	95% CI*	Category *P*-value	Variable *P*-value
Adult (>18 months) bodyweight (kg)	<10 kg	555 (49.33)	177,905 (23.72)	Base			**<0.001**
	10.0- < 15.0 kg	364 (32.47)	96,663 (12.89)	1.21	1.06–1.38	**0.005**	
	15.0- < 20.0 kg	55 (4.91)	62,265 (8.30)	0.28	0.22–0.37	**< 0.001**	
	20.0- < 25.0 kg	47 (4.19)	53,253 (7.10)	0.28	0.21–0.38	**<0.001**	
	25.0- < 30.0 kg	42 (3.75)	47,133 (6.28)	0.29	0.21–0.39	**<0.001**	
	30.0- < 40.0 kg	49 (4.37)	59,324 (7.91)	0.27	0.20–0.36	**<0.001**	
	> or = 40 kg	10 (0.89)	16,720 (2.23)	0.19	0.10–0.36	**<0.001**	
	No adult bodyweight recorded	1 (0.09)	236,737 (31.56)	0.00	0.00–0.01	**<0.001**	
Age (years)	<1.0	77 (6.87)	77,660 (10.35)	Base			**<0.001**
	1.0- < 2.0	110 (9.81)	89,576 (11.94)	1.24	0.93–1.66	0.150	
	2.0- < 3.0	137 (12.22)	71,291 (9.51)	1.94	1.47–2.56	**<0.001**	
	3.0- < 4.0	174 (15.52)	61,425 (8.19)	2.86	2.18–3.74	**<0.001**	
	4.0- < 5.0	118 (10.53)	59,788 (7.97)	1.99	1.49–2.65	**<0.001**	
	5.0- < 6.0	108 (9.63)	54,744 (7.30)	1.99	1.49–2.67	**<0.001**	
	6.0- < 7.0	125 (11.15)	51,741 (6.90)	2.44	1.83–3.24	**<0.001**	
	7.0- < 8.0	90 (8.03)	48,498 (6.47)	1.87	1.38–2.54	**<0.001**	
	8.0- < 9.0	75 (6.69)	44,905 (5.99)	1.68	1.23–2.32	**0.001**	
	9.0- < 10.0	52 (4.64)	40,479 (5.40)	1.30	0.91–1.84	0.149	
	10.0- < 11.0	23 (2.05)	36,038 (4.81)	0.64	0.40–1.03	0.064	
	11.0- < 12.0	20 (1.78)	30,950 (4.13)	0.65	0.40–1.07	0.088	
	12.0- < 13.0	8 (0.71)	26,353 (3.51)	0.31	0.15–0.63	**0.001**	
	13.0- < 14.0	2 (0.18)	20,374 (2.72)	0.10	0.02–0.40	**0.001**	
	>14.0	2 (0.18)	30,319 (4.04)	0.07	0.02–0.27	**<0.001**	
Sex-neuter status	Female entire	172 (16.49)	197,489 (26.56)	Base			**<0.001**
	Female neutered	372 (35.67)	159,622 (21.47)	2.68	2.23–3.20	**<0.001**	
	Male entire	180 (17.26)	220,815 (29.70)	0.94	0.76–1.15	0.535	
	Male neutered	319 (30.58)	165,596 (22.27)	2.21	1.84–2.66	**<0.001**	

Case no.: 1,121 referral MUO cases. Control no.: 750,00 dogs under UK primary veterinary care in 2019 in the VetCompass Programme. Column percentages shown in brackets. *P*-values < 0.050 are bolded. *CI confidence interval.

Breed-specific age distributions were summarized separately for MUO cases and non-affected dogs in the VetCompass control population. Across the most represented breeds, mean age at diagnosis in MUO cases by breed was broadly similar to the mean average age observed in the control population for Pugs (MUO cases: 3.25 years; control population: 4.08), French Bulldogs (3.31; 2.50), Yorkshire Terriers (6.85; 7.18), and Chihuahuas (4.47; 4.74). In English Springer Spaniels (6.04; 7.01), Jack Russell Terriers (6.42; 8.19), and West Highland White Terriers (6.62; 8.91), MUO was diagnosed at a younger age compared to the breed-specific mean age average (Supplementary File S1).

All study variables (breed, breed purity, Kennel Club recognized breed, Kennel Club breed group, skull conformation, brachycephalic severity, adult (>18 months) bodyweight (kg), age (years), and sex-neuter status) were liberally associated with MUO diagnosis in univariable logistic regression modelling and evaluated using multivariable logistic regression modelling. The final breed-focused multivariable model retained three risk factors: breed, age and sex-neuter status (Table 5). No biologically significant interactions were identified. The Hosmer-Lemeshow test showed no evidence of poor model fit (*p* value = 0.310). McKelvey and Zavoina’s R2 value of 0.328 showed that 32.8% of the total variance was explained by the risk factors in the model. The final model showed good discrimination (area under the ROC curve: 0.7950).

**Table 5:** Breed-focused model using multivariable logistic regression results for risk factors for meningoencephalitis of unknown origin (MUO) in dogs in the UK.

Risk factor	Category	Odds ratio	95% CI*	Category *P*-value	Variable *P*-value
Breed	Crossbreed	Base	~		**<0.001**
	Welsh Springer Spaniel	23.76	10.37–54.43	**<0.001**	
	Maltese	20.53	14.53–29.01	**<0.001**	
	Papillon	17.48	7.66–39.91	**<0.001**	
	Boston Terrier	17.31	11.17–26.82	**<0.001**	
	French Bulldog	9.14	7.14–11.71	**<0.001**	
	Dachshund	8.58	4.84–15.20	**<0.001**	
	Miniature Poodle	6.68	3.51–12.73	**<0.001**	
	Pug	6.34	4.69–8.56	**<0.001**	
	Chihuahua	6.17	4.82–7.89	**<0.001**	
	West Highland White Terrier	5.49	3.85–7.82	**<0.001**	
	Pomeranian	4.86	2.89–8.17	**<0.001**	
	Tibetan Terrier	4.63	2.04–10.52	**<0.001**	
	Cavalier King Charles Spaniel	2.82	1.83–4.36	**<0.001**	
	Bichon Frise	2.81	1.72–4.6	**<0.001**	
	Lhasa Apso	2.78	1.66–4.67	**<0.001**	
	Hungarian Vizsla	2.77	1.13–6.76	**0.026**	
	Yorkshire Terrier	2.75	1.86–4.07	**<0.001**	
	Border Terrier	2.48	1.43–4.31	**0.001**	
	Greyhound	2.34	1.19–4.61	**0.014**	
	Miniature Schnauzer	2.31	1.31–4.08	**0.004**	
	Boxer	2.17	1.10–4.26	**0.025**	
	Jack Russell Terrier	2.10	1.49–2.95	**<0.001**	
	Shih-tzu	1.76	1.18–2.63	**0.005**	
	Rottweiler	1.75	0.71–4.27	0.221	
	English Springer Spaniel	1.65	1.02–2.67	**0.040**	
	Miniature Dachshund	1.61	0.82–3.16	0.169	
	Lurcher	1.42	0.63–3.22	0.399	
	Cavapoo	1.38	0.56–3.37	0.481	
	Beagle	1.31	0.61–2.80	0.488	
	Whippet	1.30	0.48–3.52	0.605	
	Golden Retriever	1.11	0.52–2.37	0.786	
	Border Collie	1.07	0.63–1.83	0.792	
	Breed not recorded	~	~	~	
	Labradoodle	1.00	0.44–2.26	0.995	
	Purebred - other	0.94	0.69–1.28	0.695	
	Labrador Retriever	0.84	0.56–1.24	0.375	
	Cockapoo	0.78	0.46–1.30	0.340	
	English Cocker Spaniel	0.56	0.32–0.99	**0.046**	
	Staffordshire Bull Terrier	0.51	0.27–0.97	**0.042**	
	Husky	0.49	0.12–1.99	0.320	
	German Shepherd Dog	0.47	0.19–1.14	0.095	
	English Bulldog	0.41	0.10–1.66	0.211	
Age (years)	<1.0	Base	~		**< 0.001**
	1.0- < 2.0	1.00	0.73–1.36	0.997	
	2.0- < 3.0	1.42	1.05–1.91	**0.022**	
	3.0- < 4.0	1.76	1.31–2.36	**<0.001**	
	4.0- < 5.0	1.18	0.86–1.61	0.314	
	5.0- < 6.0	1.13	0.82–1.57	0.444	
	6.0- < 7.0	1.34	0.97–1.84	0.072	
	7.0- < 8.0	1.00	0.72–1.41	0.980	
	8.0- < 9.0	0.93	0.65–1.33	0.701	
	9.0- < 10.0	0.73	0.50–1.08	0.114	
	10.0- < 11.0	0.34	0.21–0.57	**<0.001**	
	11.0- < 12.0	0.40	0.24–0.66	**<0.001**	
	12.0- < 13.0	0.16	0.07–0.36	**<0.001**	
	13.0- < 14.0	0.06	0.01–0.24	**<0.001**	
	>14.0	0.04	0.01–0.16	**<0.001**	
Sex-neuter status	Female entire	Base	~		**< 0.001**
	Female neutered	4.01	3.3–4.87	**<0.001**	
	Male entire	0.98	0.80–1.21	0.877	
	Male neutered	2.98	2.44–3.63	**<0.001**	

Case no.: 1,121 referral MUO cases. Control no.: 750,00 dogs under UK primary veterinary care in 2019 in the VetCompass Programme. Column percentages shown in brackets. *P*-values < 0.050 are bolded. *CI confidence interval.

After accounting for the effects of the other variables evaluated in the multivariable modelling, 24 breeds showed increased odds of MUO compared with crossbred dogs. The breeds with the highest odds ratios included Welsh Springer Spaniel (OR 23.76, 95% CI 10.37–54.43), Maltese (OR 20.53, 95% CI 14.53–29.01), Papillon (OR 17.48, 95% CI 7.66–39.91), Boston Terrier (OR 17.31, 95% CI 11.17–26.82) and French Bulldog (OR 9.14, 95% CI 7.14–11.71). Two breeds showed reduced odds of MUO compared with crossbreds: English Cocker Spaniel (OR 0.56, 95% CI 0.32–0.99) and Staffordshire Bull Terrier (OR 0.51, 95% CI 0.27–0.97) (Table 5). Compared with dogs aged under 1 year, the odds of MUO diagnosis dropped statistically significantly once dogs reached 10 years of age. Neutered dogs showed statistically significantly higher odds of MUO than entire dogs (Table 5).

As described in the methods, breed-related variables were introduced individually to replace breed in the final breed-focused model. Compared with crossbred dogs, purebred dogs had 2.43 times higher odds (95% CI 2.03–2.91) of MUO. The toy group was the Kennel Club breed group with the highest odds (OR 5.23, 95% CI 4.36–6.27) compared to breeds that were not recognized by the Kennel Club. Breeds with brachycephalic skull conformation had 2.56 times the odds (95% CI 2.23–2.95) of MUO compared with breeds with mesocephalic skull conformation. Among dogs with brachycephaly, breeds classified with moderate (OR 1.90, 95% CI 1.24–2.90) and severe brachycephaly (OR 2.00, 95% CI 1.32–3.02) had higher odds of MUO compared to breeds with mild brachycephaly. Dogs weighing 15 kg and over had statistically lower odds of MUO compared to dogs weighing under 10 kg (Table 6).

**Table 6:** Variables that replaced breed in multivariable logistic regression modelling that also accounted for age (years) and sex-neuter status for risk factors for meningoencephalitis of unknown origin (MUO) in dogs in the UK.

Risk factor	Category	Odds ratio	95% CI*	Category *P*-value	Variable *P*-value
Breed purity	General crossbred	Base	~		**<0.001**
	Designer crossbreed	0.88	0.62–1.27	0.505	
	Purebred	2.43	2.03–2.91	**<0.001**	
Kennel club recognized breed	Not recognized	Base	~		**<0.001**
	Recognized	2.62	2.23–3.08	**< 0.001**	
Kennel club breed group	Not Kennel Club recognized breed	Base	~		**<0.001**
	Toy	5.23	4.36–6.27	**<0.001**	
	Utility	3.98	3.27–4.83	**<0.001**	
	Terrier	2.06	1.63–2.60	**<0.001**	
	Hound	1.93	1.39–2.68	**<0.001**	
	Working	1.23	0.78–1.93	0.375	
	Gundog	1.12	0.87–1.44	0.370	
	Pastoral	0.88	0.57–1.35	0.553	
Skull conformation	Mesocephalic	Base	~		**<0.001**
	Brachycephalic	2.56	2.23–2.95	**<0.001**	
	Dolichocephalic	0.85	0.65–1.10	0.225	
Brachycephalic severity	Mild	Base	~		**<0.001**
	Moderate	1.90	1.24–2.90	**0.003**	
	Severe	2.00	1.32–3.02	**0.001**	
Adult (>18 months) bodyweight (kg)	<10 kg	Base	~		**<0.001**
	10.0- < 15.0 kg	1.09	0.95–1.26	0.205	
	15.0- < 20.0 kg	0.26	0.20–0.35	**<0.001**	
	20.0- < 25.0 kg	0.30	0.22–0.40	**<0.001**	
	25.0- < 30.0 kg	0.28	0.20–0.39	**<0.001**	
	30.0- < 40.0 kg	0.28	0.21–0.38	**<0.001**	
	> or = 40 kg	0.19	0.10–0.37	**<0.001**	

Case no.: 1,121 referral MUO cases. Control no.: 750,00 dogs under UK primary veterinary care in 2019 in the VetCompass Programme. Column percentages shown in brackets. *P*-values < 0.050 are bolded. *CI confidence interval.

## 4 Discussion

### 4.1 MUO incidence risk at referral in UK

Although MUO is recognized as one of the major causes of CNS inflammation in dogs in the UK (41), diagnosis typically requires referral-level investigations and the clinical expertise of veterinary neurologists. As a result, most published studies on MUO have relied solely on cases and non-cases from (neurology) referral centers, introducing selection bias and limiting the generalizability of findings on incidence and risk factors, beyond this context. Even within referral populations, and in the absence of universally agreed criteria for antemortem diagnosis, case definitions may vary between regions, referral centers and individual specialists. These limitations underline the highly varying incidence values reported in the literature, making estimates difficult to interpret and compare due to differences in denominators, inclusion criteria, and study scope (20, 23, 35, 36, 40, 41). Although the current study is also subject to similar limitations, we tried to mitigate these by combining case selection from multiple referral centers across the UK, and using a large primary care dataset for the risk factor analysis to represent the potential underlying denominator population from which the referral cases were sourced.

Cases diagnosed with MUO represented 2.21% (95% CI: 2.08–2.34) of new neurological referrals in the current largest UK-based study investigated to date. This proportion of MUO cases referred to neurology services remained stable throughout the five-year study period (2017 to 2021). This estimate provides a practical reference to gauge the potential availability of cases for clinical trials and research.

### 4.2 A picture of antemortem MUO diagnosis in referral setting

Diagnosis of MUO during its clinical phase is challenging due to the absence of specific MUO biomarkers (32). Most MUO diagnoses, should therefore be considered as presumptive, based on clinical history, MRI findings, CSF analysis, and exclusion of infectious causes (1). However, variability in the interpretation and application of these diagnostic data may contribute to inconsistencies in MUO case definition among specialists, leading to potential under- or overclassification (1). MRI and CSF analysis are considered essential tools for diagnosing inflammatory brain diseases and more specifically MUO (54). However, a normal MRI or CSF analysis does not exclude the possibility of MUO (38). Likewise, reliance on MRI alonemay lead to false-positive diagnosis misclassification, as certain imaging findings can be indistinguishable from other conditions such as neoplasia or infectious central nervous system diseases (55). In case of suspected raised intra-cranial pressure and therefore higher risks associated with patient safety, a decision to omit CSF collection may be clinically justifiable (56). Likewise, while the exclusion of infectious disease represents a key element in the antemortem diagnosis of MUO, some variability in MUO case definition is inevitable. This consideration becomes particularly relevant in clinical scenarios where delaying treatment to await infectious disease test results may compromise patient outcomes. In such cases, clinicians may opt to initiate empirical MUO immunosuppressive therapy without pursuing infectious disease testing, relying instead on therapeutic response to guide ongoing management.

While the case definition used for MUO should promote high sensitivity and specificity in the context of clinical trials (13, 32) the application of MUO case definition in practice is poorly documented. Therefore, the current study aimed to document how veterinary specialists in UK define MUO cases based on the interpretation of diagnostic findings and the availability of diagnostic results in clinical practice. Among the case population, the majority (91.5%) of MUO diagnoses included results from both MRI and, CSF analysis, while in 8.5%, only one of these tests were performed or, in a few cases, CT was performed instead of MRI (Figure 1). CSF was more commonly omitted due to patient safety concern, in the risk of brain herniation, while financial constraints were the primary reason for not performing MRI scans.

For MUO cases that underwent both MRI and CSF sampling, results suggestive of MUO for both MRI and CSF analysis were observed in ~90% of cases; with just 3.5% of MUO diagnoses showing normal MRI and abnormal CSF results, and 5.4% having normal CSF results despite detectable lesions on MRI studies. It has been previously estimated that approximately 25% of brain MRI scans in dogs with inflammatory CSF may show no abnormalities (14, 38, 57) with later reports indicated that up to 7% of dogs with MUO present with a normal MRI (2, 13), aligning with the findings of the current study. Likewise, the current CSF analysis results are consistent with previous studies, which have reported normal findings in 3–57% of inflammatory CNS diseases (13, 58–60). In the current study case load, only 1.9% of dogs were diagnosed with MUO despite having unremarkable MRI and CSF results (Figure 1). In these cases, the clinicians still diagnosed MUO based on clinical history and/or response to immunosuppressive treatment.

Recommendations for antemortem MUO diagnosis require ruling out active central nervous system infections based on their geographic prevalence and perceived risk (1, 13, 21, 32, 57). Ideally, broader prospective screening, including viral, bacterial, fungal, and parasitic agents, should be considered to more comprehensively rule out infectious causes. The selection of pathogens for screening should also be informed by relevant epidemiological data. In the UK, *Neospora caninum* and *Toxoplasma gondii* are considered the most common infectious causes of canine encephalitis. However, reporting of multiple cases of tick-borne encephalitis (TBE) caused by flavivirus in very recent years (61) highlights the need for further research into TBE epidemiology. Future UK screening protocols for infectious agents may therefore also expand to include TBE. Among the MUO diagnoses in this study, infectious agent screening was limited to the exclusion of *Toxoplasma gondii* and *Neospora caninum*, with less than 10% of MUO cases not tested for either agent at the time of diagnosis. Coelho et al. (62) identified a prevalence of active infection with *Toxoplasma gondii* and *Neospora caninum* at 0.25 and 2.25%, respectively, in a large cohort of MUO dogs in the UK. However, given that some MUO cases in the current study were not tested suggests that, in certain situations, specialists may not always consider the exclusion of protozoal disease essential, possibly due to economic considerations, practical constraints or a perceived low risk.

Despite reported utility of brain biopsy for a more definitive antemortem diagnosis of MUO (63, 64), none of the dogs in this dataset underwent the procedure. This likely reflects both inherent procedural challenges, cost, associated risk and accumulating evidence (26), that, for this subset of encephalopathies, local brain biopsy may lack diagnostic accuracy, as it might fail to capture the widespread inflammatory process across the whole brain. Accordingly, the current study shows that biopsy is not routinely incorporated into the diagnostic workup for MUO, even in specialist settings, as previously reported (21). Likewise, no electroencephalography data was available for review for this subset of patients.

Nonetheless, while our data do indicate a tendency among specialists to aim for the most accurate diagnosis in the MUO cases that are diagnosed, there still remains some variation in MUO case definition and diagnostic criteria. This variation appears driven by clinical and financial constraints and, at times, emergency considerations, underscoring the complexities of real-world clinical decision-making in MUO diagnosis. Although proposed guidelines for standardizing the diagnostic criteria for MUO clinical trials, in the absence of histopathological confirmation have been previously published (13), it remains unclear how consistently these criteria can be applied in clinical settings. Of the 1,121 MUO cases in the current study, 868 (77.43%) retrospectively fulfilled all four proposed eligibility criteria for therapeutic trials as outlined by Granger et al., including age above 6 months, MRI and CSF abnormalities consistent with inflammatory disease, and exclusion of active CNS infection. Our findings indicate that facilitating multicenter clinical studies to further characterize the clinical presentation, diagnostic findings, treatment outcomes, and prognosis in dogs with presumed MUO requires a balanced approach. Specifically, accepting that not all diagnosed MUO cases will meet very strict inclusion criteria could enhance the generalizability of trial results, while conversely, applying highly stringent criteria may prolong recruitment times and limit case availability.

### 4.3 Demographic risk factors of MUO

Most previously available studies reporting the demographic data on MUO in dogs are based solely on referral center populations. Referral caseloads are recognized to represent a pre-selected clinical subset that may be poorly representative of the general dog population (65). Analyses that compare referral cases to an overall underlying referral population therefore may not accurately reflect broader demographic variables, introducing additional geographic and cultural biases (13). To help mitigate these issues from referral-only data analysis and to enhance generalizability of results, the current study used a case–control approach to compare a large referral case population of 1,121 dogs with a diagnosis of MUO against a control group of 750,000 dogs randomly sampled from dogs registered in primary care veterinary practices across the UK within VetCompass (46, 66).

Seventy-five distinct breeds were identified among MUO cases. Of these, 24 showed significantly increased MUO odds compared to crossbred dogs. These findings align with existing literature (1, 13, 21), reinforcing that while MUO can potentially affect any breed, some specific breeds are highly predisposed and also that being a purebred dog overall is a risk factor. Among MUO cases, French Bulldogs and Chihuahuas were the most represented breeds numerically (Table 7), but this apparent over-re-presentation reflects the combined effects of breed popularity in the wider dog population as well as also accounting for individual breed risk. After accounting for breed population sizes, the risk factor analysis revealed that Maltese (OR 20.53, 95% CI 14.53–29.01), Papillon (OR 17.48, 95% CI 7.66–39.91), and Boston Terrier (OR 17.31, 95% CI 11.17–26.82) had highest odds (Table 4). Interestingly, Welsh Springer Spaniels exhibited the overall highest odds ratio for MUO (OR 23.76, 95% CI 10.37–54.43) (Table 4). Despite their high relative risk, this breed has not been previously reported as highly affected in the literature so this novel association, while statistically robust, warrants confirmation in later studies.

**Table 7:** Descriptive and univariable logistic regression results for breed as a risk factor for meningoencephalitis of unknown origin (MUO) in dogs in the UK.

Breed	Case No. (%)	Control No. (%)	Odds ratio	95% CI	Category *P*-value	Variable *P*-value
Crossbreed	152 (13.56)	179,231 (23.9)	Base			**<0.001**
Maltese	45 (4.01)	2,871 (0.38)	18.48	13.23–25.83	**<0.001**	
Welsh Springer Spaniel	6 (0.54)	383 (0.05)	18.47	8.12–42.02	**<0.001**	
Boston Terrier	26 (2.32)	1781 (0.24)	17.21	11.33–26.16	**<0.001**	
Papillon	7 (0.62)	614 (0.08)	13.44	6.28–28.80	**<0.001**	
French Bulldog	135 (12.04)	22,355 (2.98)	7.12	5.65–8.98	**<0.001**	
Dachshund	13 (1.16)	2,169 (0.29)	7.07	4.00–12.47	**<0.001**	
Pug	67 (5.98)	13,376 (1.78)	5.91	4.43–7.88	**<0.001**	
Miniature Poodle	10 (0.89)	2,150 (0.29)	5.48	2.89–10.41	**<0.001**	
Chihuahua	122 (10.88)	26,596 (3.55)	5.41	4.26–6.87	**<0.001**	
Tibetan Terrier	6 (0.54)	1,611 (0.21)	4.39	1.94–9.94	**<0.001**	
Pomeranian	18 (1.61)	4,953 (0.66)	4.29	2.63–6.99	**<0.001**	
West Highland White Terrier	43 (3.84)	11,903 (1.59)	4.26	3.03–5.98	**<0.001**	
Bichon Frise	20 (1.78)	8,400 (1.12)	2.81	1.76–4.48	**<0.001**	
Greyhound	9 (0.80)	4,283 (0.57)	2.48	1.26–4.86	**0.008**	
Hungarian Vizsla	5 (0.45)	2,374 (0.32)	2.48	1.02–6.06	**0.046**	
Cavalier King Charles Spaniel	24 (2.14)	11,618 (1.55)	2.44	1.58–3.75	**<0.001**	
Lhasa Apso	17 (1.52)	8,199 (1.09)	2.44	1.48–4.04	**<0.001**	
Miniature Schnauzer	13 (1.16)	6,965 (0.93)	2.20	1.25–3.88	**0.006**	
Yorkshire Terrier	33 (2.94)	17,662 (2.35)	2.20	1.51–3.21	**<0.001**	
Border Terrier	14 (1.25)	8,348 (1.11)	1.98	1.14–3.42	**0.015**	
Boxer	9 (0.80)	5,829 (0.78)	1.82	0.93–3.57	0.081	
Jack Russell Terrier	49 (4.37)	33,925 (4.52)	1.70	1.23–2.35	**0.001**	
English Springer Spaniel	24 (2.14)	17,337 (2.31)	1.63	1.06–2.51	**0.026**	
Shih-tzu	29 (2.59)	22,490 (3.00)	1.52	1.02–2.26	**0.039**	
Cavapoo	6 (0.54)	4,687 (0.62)	1.51	0.67–3.41	0.323	
Whippet	5 (0.45)	4,086 (0.54)	1.44	0.59–3.52	0.420	
Miniature Dachshund	10 (0.89)	8,303 (1.11)	1.42	0.75–2.69	0.283	
Rottweiler	5 (0.45)	4,368 (0.58)	1.35	0.55–3.29	0.510	
Lurcher	6 (0.54)	5,334 (0.71)	1.33	0.59–3.00	0.498	
Labradoodle	8 (0.71)	7,235 (0.96)	1.30	0.64–2.66	0.465	
Beagle	7 (0.62)	6,589 (0.88)	1.25	0.59–2.67	0.560	
Golden Retriever	8 (0.71)	9,127 (1.22)	1.03	0.51–2.10	0.928	
Breed not recorded	0 (0.00)	4,493 (0.60)	~			
Cockapoo	20 (1.78)	24,206 (3.23)	0.97	0.61–1.55	0.913	
Border Collie	15 (1.34)	20,769 (2.77)	0.85	0.50–1.45	0.553	
Purebred - other	63 (5.62)	89,174 (11.89)	0.83	0.62–1.12	0.223	
Labrador Retriever	31 (2.77)	51,595 (6.88)	0.71	0.48–1.04	0.080	
English Cocker Spaniel	19 (1.69)	32,138 (4.29)	0.70	0.43–1.12	0.138	
Husky	3 (0.27)	5,780 (0.77)	0.61	0.20–1.92	0.400	
German Shepherd Dog	6 (0.54)	15,853 (2.11)	0.45	0.20–1.01	0.053	
Staffordshire Bull Terrier	11 (0.98)	31,198 (4.16)	0.42	0.23–0.77	**0.005**	
English Bulldog	2 (0.18)	7,642 (1.02)	0.31	0.08–1.25	0.099	

Case no. 1,121 referral MUO cases. Control no.: 750,00 dogs under UK primary veterinary care in 2019 in the VetCompass Programme. Column percentages shown in brackets. *P*-values < 0.050 are bolded. *CI confidence interval.

Among breeds with higher bodyweight, Springer Spaniels and Labrador Retrievers appeared numerically overrepresented in the MUO group matching previous findings from a UK based dataset (3). However, after adjusting for breed population sizes in the wider primary care dog population, Labrador Retrievers did not show increased odds relative to crossbreeds, while English Springer Spaniels demonstrated a modestly increased odds of MUO diagnosis compared to crossbreeds (OR 1.65, 95% CI 1.02–2.67) (Table 4). The high representation of Labrador Retrievers in our group of cases and in previous reports likely reflects their popularity in the UK rather than a true heightened risk for MUO. Meanwhile, the overrepresentation of English Springer Spaniels appears to stem from both their modestly increased odds and high ownership within the general population. This observation reinforces the need to account for breed distribution by using multivariable risk factor analysis when evaluating disease risks rather than just counting case numbers from descriptive studies (66).

Potential genetic risk factors of MUO have been investigated in some commonly affected breeds. Genes involved in immune system regulation and T-cell development (ILR7 on chromosome 4) and tumor suppressor associated with cell cycle regulation (FBXW7 on chromosome 15), are suspected to play a role in the development of necrotizing encephalitis (NE), a subtype of MUO, in Maltese dogs (67). A relative risk of 5.45 of developing necrotizing meningoencephalitis (NME) has been reported in Pugs carrying specific MHC class II alleles on chromosome 12 compared to Pugs not carrying those alleles, for which a commercial genetic test is available (15). A recent study has identified two specific genetic markers, a DLA-DRB1 015:01–DQA1 006:01–DQB1 023:01 haplotype and the DLA-DQB1 023:01 allele associated with increased risks for MUO in Chihuahuas (68).

These breed-dependent haplotypes point to distinct immunogenetic mechanisms that may underlie disease development across different canine populations. Such variation, could account for differences in clinical presentation, response to treatment, and disease progression observed in breed-specific cohorts, as reported previously for Pugs (9), Yorkshire Terriers (2), and more recently French Bulldogs (69). Our findings may therefore support future research on MUO phenotypes and immunogenetic risk factors in additional breeds, including Papillons, Boston Terriers, French Bulldogs, and Welsh Springer Spaniels.

The structured case–control model used in our study, aimed to identify breeds with possible genetic protection against MUO compared to crossbred dogs. In the current risk analysis, the English Cocker Spaniel (OR 0.56) and Staffordshire Bull Terrier (OR 0.51,) exhibited reduced odds of MUO diagnosis compared to crossbred dogs. Follow-up comparative studies involving low- and high-risk breeds may be considered, to help uncover allelic variants associated with both susceptibility and protection.

Current multivariable analysis’s results also highlights interplay between morphology, sex-neutered status and age at presentation for risk of MUO diagnosis. Dogs with brachycephalic skull conformation overall had 2.56 times the odds of MUO diagnosis compared with mesocephalic breeds. Among those dogs with brachycephaly, dogs with extreme brachycephaly showed 2.00 times the odds of MUO diagnosis compared with dogs with mild brachycephaly. In additions, some individual breeds with extreme brachycephaly showed concerningly high odds of MUO diagnosis compared with crossbred dogs: Boston Terrier had 17.31 times the odds and French Bulldog had 9.14 times the odds (Table 4).

A French Bulldog predisposition to neurological disorders in general has been previously suggested (70), with a possible link to their extreme skull conformation. For brachycephalic breeds, links between genetic markers such as Fibroblast Growth Factor 4 (FGF4), SPARC-related Modular Calcium Binding Gene 2 (SMOC2), and DISHEVELLED 2 (DVL2) have been established, respectively, with intervertebral disc herniation and Brachycephalic Obstructive Airway Syndrome (71–73). While no direct association between MUO and genetic markers linked to brachycephalic traits has been identified so far, our findings suggest the need to explore these genetic and morphological overlaps further. For example, CSF anomalies and hypoxia, conditions already linked to brachycephaly (74), —may compromise blood–brain barrier integrity against inflammatory molecules and cells, a proposed factor in MUO pathogenesis (32, 75, 76). However, while brachycephaly emerges as a significant morphological risk factor in our analysis, several non-brachycephalic breeds also showed a high MUO risk. This suggests that other breed-related factors beyond abnormal skull morphology, such as genetic or immunological traits, may contribute in MUO predisposition. Nonetheless, brachycephaly could act as an exacerbating factor, further reinforcing hypotheses on a multifactorial aetiopathogenesis of the disease (44, 77).

There is a large body of previous work reporting that small- and toy-breed dogs are overrepresented among MUO cases (1, 5, 21, 44). The current study supports a strong association between smaller body mass and raised MUO risk, with toy KC dogs showing the highest risk among the KC breed groups, dogs >15 kg exhibiting progressively lower odds, and the >40 kg group having the lowest risk (OR 0.19) (Table 7). While the present data indicate a reduced MUO risk in larger breeds, the underlying mechanisms remain unclear. One speculative hypothesis could be that cranial miniaturization in small and toy breeds may result in reduced skull capacity relative to brain volume, potentially contributing intracranial crowding or altered cerebrospinal fluid dynamics (78, 79). However, an apparent protective effect in larger breeds also raises further hypotheses involving immunological, genetic, metabolic, or physiological differences between size groups in their susceptibility to MUO.

Our multivariable results also support previous findings (1, 13, 21, 23) that MUO is a diagnosis of younger dogs, with a peak incidence in young adults between 3 and 4 years of age, and a median age of 4.33 years. However, two distinct breed-related patterns emerged: Pugs and French Bulldogs exhibited earlier onset, with a median age of approximately 3 years while Jack Russell Terriers, West Highland White Terriers, Yorkshire Terriers, and Springer Spaniels showed later onset (6–7 years). A statistical difference in age distribution has been previously reported between dogs affected by the MUO subtypes NE/NME and GME, with NME typically occurring in dogs under 4 years old and GME predominantly in those between 4 and 8 years old (13). These patterns, suggest that GME is more frequent in certain breeds, and that in these breeds clinical manifestations—and therefore diagnosis—tend to occur at older ages. Recently, a report on three Australian Sheperd diagnosed at 7, 10, and 11 years of age, suggests that age-dependent susceptibility, could extend to breeds beyond those traditionally recognized (29). While histopathological confirmation was beyond the scope of our study, the current findings support a demographic explanation for the observed age variation among MUO cases, rather than a biological predisposition for breed-specific early or late onset. In the control population, Jack Russell Terriers, West Highland White Terriers, Yorkshire Terriers, and Springer Spaniels had a mean age of >7 years. Similarly, for Pugs and French Bulldogs, age was similar between the control and MUO cases (<4 years) (Supplementary File S1). It is therefore plausible that the apparent difference in the age of onset for MUO does not necessarily reflect an intrinsic predisposition for a specific subset of MUO to occur earlier or later in specific breeds, rather than simply mirroring the demographics of the population of origin. This interpretation aligns with previous reports on median age variation among common UK dog breeds (80), which appear influenced by breed-specific life expectancy differences (81) and highlights the importance of accounting for confounding factors, when interpreting demographic data on MUO.

Moreover, while some of the early work on MUO had proposed that there were distinct clinical signs and histopathologic findings among MUO subtypes (9, 13), emerging evidence indicates significant overlaps in MUO histological types (26, 28), even within the same affected dog, and that previously unreported MUO subtypes may also exist (28). Together with our findings, this evidence indicating overlapping histological subtypes and the possibility of unrecognized MUO forms, highlights the need for a classification framework that better integrates clinical presentation with histopathological patterns and risk factors.

The current multivariable analysis showed a significant drop in MUO diagnosis odds after 10 years of age, suggesting some possible protective effects of older age. Possible explanations include immune-physiological events associated to aging, environmental factors and human factors. However, this could also reflect selection bias against pursuing MUO diagnosis and treatment as dogs approach geriatric ages and instead a move toward pragmatic management in general practice without referral, or electing for euthanasia on clinical grounds without a final diagnosis being reached (82, 83). Nonetheless, MUO is still considered (44) an idiopathic self-directed immune response. It may be therefore speculated that younger dogs possess more reactive immune systems compared to older dogs, which may predispose them to autoimmune conditions like MUO. Age-related declines in naïve CD4+/CD8 + T cells and altered cytokine activity (84–86) could also lower risk in older dogs. However, current observations do not strongly support the theory that age-related immunological impairment alone could serve as a protective factor against the disease. Instead, hypotheses on MUO aetiopathogenesis increasingly point to multigenetic defects that predispose the immune response to shift from protective to noxious (10, 15, 33, 67, 68). Even so, comparative studies on immune profiling across age groups in predisposed breeds may help identify resilience biomarkers of the disease.

Some previous studies have suggested a possible female predisposition for MUO in dogs (8, 45, 87), although these studies were limited by relatively small sample sizes. Comprehensive reviews failed to identify any sex-based association with MUO risk (2, 13, 21). The current analysis based on much larger numbers of cases and controls than previous studies did not identify a significant sex predisposition for MUO diagnosis in dogs but did indicate that neutered dogs had significantly higher odds of MUO diagnosis compared to entire dogs (Table 7). However, this result on neutering requires cautious interpretation. Previous studies suggest that post-neutering hormonal changes may influence immune-mediated diseases in dogs, potentially through mechanisms such as increased oxidative stress (88, 89). However, the lack of data on neutering timing in the MUO and control populations in the current study limits conclusions. Additionally, since all dogs in the current study were under referral or primary veterinary care, this could have introduced potential bias towards, for example, owners who seek regular veterinary attention and are more likely to neuter their dogs. Therefore, while the results relating to neutering as a possible predisposing factor for MUO are interesting, large prospective studies that include the age at neutering and carefully account for other potential biases are needed before stronger conclusions can be reached on any associations between neutering and MUO.

The current study observed no seasonal pattern in MUO incidence, in agreement with prior work (20, 41) This finding supports the view that few cases of MUO are likely to be vector-borne, a hypothesis supported by a lack of association between MUO and pathogens or infectious triggers demonstrated to date (77). Non-infectious seasonal factors, such as changes in sun exposure, have also previously been proposed as modulators of immune tolerance, potentially contributing to immune-mediated diseases in both dogs and humans (90, 91). However data from studies conducted across diverse climates, including this UK-based analysis (20, 92), consistently reinforce that neither climatic variation nor seasonal vector activity appear to influence MUO disease onset or progression.

## 5 Limitations

The present study has several limitations. First, case selection was based on referred caseloads, which introduces potential selection bias, as previously discussed (65). Referral criteria may vary depending on geographic location (e.g., ease of access to specialist centers) and the habits of individual veterinarians and clinics, and as well on owner personal and economic reasons. These factors may influence which cases reach specialist care.

Additional unmeasured confounding factors may have affected the estimation of MUO incidence in the current study, as data were obtained retrospectively. For example, completeness and consistency of clinical records may have varied between institutions and over time, potentially impacting the quality and availability of extracted data.

Case identification was performed either by board-certified neurologists, supervised residents or interns at each center, or by direct review from one of the authors (YC). This strategy may have introduced variability in case selection across sites. The inclusion of 13 neurology services, each under the direction of different specialists, further contributes to potential heterogeneity. Differences in interpretation of clinical and diagnostic data among specialists may have led to inter-center variation in the number of cases classified as MUO prior to data extraction and therefore influenced the total number of cases included in the study. This may be particularly relevant to the small number of MUO diagnoses made despite normal MRI and/or CSF findings: it is unclear whether this proportion reflects a genuinely rare presentation or whether such cases were systematically excluded from MUO classification.

Although histopathological confirmation was beyond the scope of this study, the lack of tissue validation introduces inherent diagnostic uncertainty. This also implies that some cases diagnosed as MUO may have been misclassified, potentially leading to an over or underestimation of the incidence risk observed in this study.

Regarding the methods used to analyze demographic risk factors, in case–control studies the ideal situation is for controls to represent the population from which cases are derived. In the current study, MUO cases were derived from a referral population and controls from VetCompass dogs registered with primary care practices. However, all referral cases emanate from primary care and therefore it was felt more appropriate to use a control population of primary care dogs rather than a biased subset of primary care dogs that were referred for some condition other than MUO. However, it is not clear what proportion of the referred MUO cases originated specifically from the veterinary clinics included in the VetCompass control population. Nonetheless, it was considered likely that the VetCompass population represents a reasonable estimate of the background veterinary-attending population from which the cases may have originated.

The case–control population extracted from the VetCompass dataset provides information on dogs registered at veterinary practices in 2019, while MUO case records originate from a broader time frame spanning 2017 to 2021. The use of datasets covering different time periods may have introduced unmeasured confounding factors that influenced the study’s outcomes. In particular, changing trends of ownership for individual dog breeds across multiple years could have affected the findings of this study.

Finally, the retrospective nature of the current study prevented analysis of additional risk factors, aside from those routinely recorded in the clinical records, such as breed, age, and sex.

## 6 Conclusion

This study offers an updated epidemiological benchmark, demonstrating that MUO cases consistently accounted for approximately 2.21% of neurological referrals across UK specialist centers over a five-year period. This stable proportion provides a practical reference point for estimating case availability in clinical research and trial design.

Our analysis underscores the necessity of a balanced recruitment strategy for multicenter therapeutic trials. Although the proposed eligibility criteria can be retrospectively fulfilled by the majority of cases, strict adherence may hinder recruitment efficiency and compromise external validity. A flexible yet diagnostically robust approach may be warranted to optimize feasibility and inclusivity.

Finally, our findings reveal additional breed-specific risk profiles and potential protective traits, lending further support to the multifactorial aetiopathogenesis of MUO involving immunogenetic, morphologic, and age-related components. These results advocate for future investigations into novel breed-specific genetic susceptibility, protective biomarkers, and mechanisms of immune resilience to advance the understanding and management of MUO.

## Data Availability

The original contributions presented in the study are included in the article/Supplementary material, further inquiries can be directed to the corresponding author.
